# Efficacy and safety of weekly *nab*-paclitaxel plus gemcitabine in Chinese patients with metastatic adenocarcinoma of the pancreas: a phase II study

**DOI:** 10.1186/s12885-017-3887-z

**Published:** 2017-12-22

**Authors:** Ruihua Xu, Xianjun Yu, Jihui Hao, Liwei Wang, Hongming Pan, Guohong Han, Jianming Xu, Yanqiao Zhang, Shujun Yang, Jia Chen, Jieer Ying, Guanghai Dai, Mingyu Li, Damir Begic, Brian Lu, Lin Shen

**Affiliations:** 1 Sun Yat-sen University Cancer Center, 651 Dongfeng East Road, Guangzhou, 510060 China; 2 Fudan University Shanghai Cancer Center, No 270, Dongan Road, Shanghai, 200032 China; 3 Tianjin Cancer Hospital, Huan-Hu-Xi Road, Tianjin, 300060 China; 4 Renji Hospital, Shanghai Jiaotong University, 160 Pujian Lu, Shanghai, 200127 China; 5 Sir Run Run Shaw Hospital, Zhejiang University, 3 East Qingchun Road, Hangzhou City, 310016 China; 6 Xijing Hospital, W Rd, Xi’an, Changle, 127 China; 7 307 Hospital of the People’s Liberation Army, Beijing, 100021 China; 8 Harbin Medical University Cancer Hospital, Haping Road No.150, Harbin, China; 9 Henan Cancer Hospital, Zhengzhou, 450003 China; 10 Jiangsu Provincial Tumor Hospital, 300 Guangzhou Road, Nanjing, 210029 China; 11 Zhejiang Cancer Hospital, 38 Guangji Road, Banshan Bridge, Hangzhou City, 310022 China; 12 Chinese People’s Liberation Army General Hospital No.28, Fuxing Road, Beijing, China; 13 Celgene Corporation, Summit, NJ USA; 14 Peking University Cancer Hospital and Institute, No. 52 Fucheng Road, Haidian District, Beijing, 100142 China; 15 Department of Gastrointestinal Oncology, Peking University Cancer Hospital and Institute, No. 52 Fucheng Road, Haidian District, Beijing, 100142 China

**Keywords:** *nab*-paclitaxel, Gemcitabine, MPACT, Pancreatic cancer, Metastatic, Chinese

## Abstract

**Background:**

This phase II bridging study assessed the safety and efficacy of *nab*-paclitaxel/gemcitabine (Metastatic Pancreatic Adenocarcinoma Clinical Trial [MPACT] regimen) in Chinese patients with metastatic pancreatic cancer (MPC).

**Methods:**

This 3-part sequential study evaluated *nab*-paclitaxel 125 mg/m^2^ plus gemcitabine 1000 mg/m^2^ on days 1, 8, and 15 every 4 weeks. Part 1 evaluated safety. Part 2 evaluated efficacy using Simon’s optimal 2-stage design: if >2 responses were observed in Stage 1 (*n* = 28), 54 additional patients would be enrolled in Stage 2. If >9 responses were observed, the study was complete. Otherwise, *nab*-paclitaxel/gemcitabine would be compared with gemcitabine alone in Part 3. The primary endpoint was overall response rate (ORR). Secondary endpoints included duration of response (DOR), overall survival (OS), and safety.

**Results:**

Eighty-three patients were treated. The prespecified primary endpoint was met: the independently assessed ORR in Stages 1 + 2 was 35% (95% CI, 24.8–46.2); therefore, Part 3 was not initiated. The median DOR was 8.9 months (95% CI, 6.01–8.94). The median OS and progression-free survival were 9.2 (95% CI, 7.6–11.1) and 5.5 (95% CI, 5.29–7.16) months, respectively. The 12-month OS rate was 30%. In an updated analysis, the median OS was 9.3 months and the 12-month OS rate was 32%. Longer OS was observed in patients with baseline neutrophil-to-lymphocyte ratio ≤ 5 vs > 5. The most common grade ≥ 3 adverse events were leukopenia (35%), neutropenia (34%), anemia (15%), thrombocytopenia (10%), and fatigue (13%). Grade 3 peripheral neuropathy occurred in 7% of patients (no grade 4 reported).

**Conclusions:**

The MPACT regimen of *nab*-paclitaxel/gemcitabine is efficacious in Chinese patients with MPC. No new safety signals were observed.

**Trial registration:**

NCT02135822, May 8, 2014.

## Background

Pancreatic cancer is a growing health problem in China, where, similar to global trends, mortality nearly equals incidence [1, 2]. Epidemiological data from China’s National Cancer Center Registry estimate that 79,400 people died from this disease in 2015 [3]. However, because these data are collected from multiple population-based cancer registries, they represent a small portion of the Chinese national population and may underestimate the true burden of pancreatic cancer. Similarly, a paucity of survival data exists for Chinese patients. A recent study from the Shanghai Cancer Registry reported a 5-year overall survival (OS) rate of 4.1% for all stages and tumor grades analyzed [4]. In China, approved treatment options for metastatic pancreatic cancer (MPC) are limited.

In the European Union and the United States, *nab*-paclitaxel in combination with gemcitabine has received approval for the first-line treatment of MPC [5, 6]. This approval was based on the global phase III Metastatic Pancreatic Adenocarcinoma Clinical Trial (MPACT), in which first-line *nab*-paclitaxel/gemcitabine treatment demonstrated a significantly better OS and overall response rate (ORR) than did gemcitabine alone in 861 patients from North America, Europe, and Australia [7, 8]. The combination of *nab*-paclitaxel/gemcitabine is also recommended for first-line treatment of patients with MPC by the National Comprehensive Cancer Network guidelines, which are often followed by Chinese physicians [9]. *nab*-Paclitaxel/gemcitabine may also be a suitable first-line treatment regimen for Chinese patients with MPC, despite known differences in cancer drug tolerability between Asian and white populations [10]. These differences may result from genetic or environmental factors, among other things, and one of the most commonly reported examples is increased chemotherapy-induced myelosuppression in Asian vs white patients [11–13]. Based on clinical trials in metastatic breast cancer, the safety profile of *nab*-paclitaxel monotherapy appears largely similar between Western and Chinese populations [14, 15]. However, limited data exist on the safety and tolerability of *nab*-paclitaxel/gemcitabine in Chinese patients. A phase I/II study evaluated this combination in Chinese patients with advanced pancreatic cancer, albeit at a dose and schedule different from that administered in MPACT [7, 8, 16]. Although the study did not meet its primary endpoint of identifying the maximum tolerated dose in Chinese patients, *nab*-paclitaxel 120 mg/m^2^ (the highest dose tested) plus gemcitabine 1000 mg/m^2^ on days 1 and 8 every 3 weeks was the recommended dosage/schedule for these patients. With respect to dose intensity, this regimen was comparable with the MPACT regimen and resulted in a tolerable safety profile [7, 16].

In this phase II study, the efficacy and safety of the *nab*-paclitaxel/gemcitabine regimen used in the MPACT study were evaluated in Chinese patients with MPC.

## Methods

### Study Population

Patients with histologically or cytologically confirmed metastatic pancreatic adenocarcinoma measurable by Response Evaluation Criteria in Solid Tumors (RECIST) version 1.0 were enrolled in this study. Key eligibility requirements included ≥18 years of age, no prior treatment for metastatic disease, Karnofsky performance status (KPS) ≥ 70, and adequate hematologic, renal, and liver function. Patients with known brain metastases or baseline peripheral neuropathy grade ≥ 2 were excluded.

This study was conducted in accordance with the Declaration of Helsinki and Good Clinical Practice Guidelines of the International Conference on Harmonisation. Informed consent was obtained from all patients prior to study entry. The trial is registered at ClinicalTrials.gov (NCT02135822).

### Study Design

This phase II, multicenter, 3-part sequential study was conducted at 13 sites in China. Part 1 evaluated the dose of *nab*-paclitaxel/gemcitabine based on safety. In Part 1, 10 patients were to be enrolled and treated with *nab*-paclitaxel 125 mg/m^2^ intravenously (IV) plus gemcitabine 1000 mg/m^2^ IV once weekly for 3 weeks followed by a week of rest (qw 3/4). Safety data were evaluated after the last enrolled patient completed 2 treatment cycles or earlier if treatment was not tolerable or when ≥66% of patients tolerated ≥2 treatment cycles without dose delay or modification. If it was determined in Part 1 that *nab*-paclitaxel 125 mg/m^2^ was the recommended dose for Part 2, the 10 patients from Part 1 were counted as a portion of the Part 2 enrollment. If the initial dose level in Part 1 was not tolerated, the Part 2 starting doses were to be reduced to *nab*-paclitaxel 100 mg/m^2^ plus gemcitabine 800 mg/m^2^.

Part 2 evaluated the efficacy of *nab*-paclitaxel/gemcitabine based on a single-arm, Simon’s optimal 2-stage design [17]. Patients in Part 2 were treated with the *nab*-paclitaxel and gemcitabine dose levels selected from Part 1. In Stage 1, the planned enrollment was 28 patients. If >2 responses were observed, an additional 54 patients would be enrolled in Stage 2 for treatment at the same dose level. In Stage 2, if >9 of 82 responses were observed, the study would be complete. If an insufficient number of responses was observed after Stage 1 or Stage 2, the study would progress to Part 3.

Part 3 was designed to evaluate the efficacy and safety of *nab*-paclitaxel/gemcitabine vs gemcitabine alone based on a randomized 2-arm design. Planned total enrollment for Part 3 was 154 patients. Patients were to be randomized 1:1 to receive the Part 1 recommended dose of *nab*-paclitaxel followed by gemcitabine on days 1, 8, 15, 29, 36, and 43 or gemcitabine 1000 mg/m^2^ IV alone weekly for 7 of 8 weeks (cycle 1). Subsequent treatments in both arms would occur on days 1, 8, and 15 of a 28-day cycle. Randomization would be stratified by liver metastasis and KPS score.

### Study Assessment

The primary endpoint of the study was independently assessed ORR according to RECIST 1.0. Secondary endpoints included duration of response (DOR) according to RECIST 1.0, OS, safety, and tolerability. Exploratory endpoints were disease control rate (the percentage of patients achieving objective tumor response or stable disease for ≥16 weeks), serum carbohydrate antigen 19–9 levels and potential association with clinical outcomes, patient-reported quality of life using the European Organisation of Research and Treatment of Cancer Quality of Life Questionnaire-Core 30, and tumor biomarker analysis. Ad hoc analyses included progression-free survival (PFS) and potential association of baseline neutrophil-to-lymphocyte ratio (NLR) and OS. Efficacy was evaluated in the intent-to-treat population, which included all enrolled patients. Response and progression were independently assessed by a central imaging reviewer, blinded to treatment, according to radiological review by computed tomography scan or magnetic resonance imaging every 8 weeks per RECIST 1.0. Treatment continued until unacceptable toxicity or disease progression. Safety was assessed on days 1, 8, 15, and 22 of each cycle by the investigator in all patients who received ≥1 dose of study drug. Adverse events (AEs) were classified by the Medical Dictionary for Regulatory Activities version 17.0 system, and severity was evaluated according to the National Cancer Institute’s Common Terminology Criteria for Adverse Events version 3.0. Dose reductions, delays, premature discontinuations, and clinical laboratory data were also evaluated.

### Sample Size and Statistical Analysis

In Part 2, Simon’s optimal 2-stage design was used. The 1-sided hypothesis test on the ORR was H_0_: ORR ≤ 7% vs H_1_: ORR ≥ 19%. The hypotheses were based on the ORR results from MPACT; the observed ORR was 23% (2-sided 95% CI, 19%–27%) for the *nab*-paclitaxel/gemcitabine arm and 7% (2-sided 95% CI, 5%–10%) for gemcitabine alone. The planned sample size of 82 patients was estimated to provide 90% power at a 1-sided significance level of 0.05 [7]. The primary endpoint was analyzed based on the exact binomial distribution, and a 2-sided 95% CI was estimated using the Clopper-Pearson method. DOR, OS, OS by baseline NLR (cutoffs = 5 and median value), and PFS were analyzed by the Kaplan-Meier method. The data cutoff date was 1 June 2015. Data obtained using a cutoff date of 9 June 2016 were analyzed to determine updated OS rates. For the OS by baseline NLR subgroup analysis, the hazard ratio (HR) and 2-sided 95% CI were estimated using the nonstratified Cox proportional hazard model, and the survival distributions for the 2 baseline NLR groups were compared using the nonstratified log-rank test.

## Results

### Patients

In total, 83 patients were enrolled in Part 2. The baseline characteristics are described in Table 1. The median age was 57.0 years, and 19% of patients were aged ≥65 years. Most patients (70%) had a baseline KPS of 90 to 100. The median baseline carbohydrate antigen 19–9 level for all patients was 602.8 U/mL.

**Table 1:** Baseline characteristics

Patient characteristics	*N* = 83
Age, median (range), years	57.0 (30–78)
≥ 65 years, %	19
Male, %	70
KPS, %	
90–100	70
70–80	30
Current site(s) of metastasis, %	
Hepatic/liver	83
Abdomen/peritoneal	53
Lung/thoracic	18
No. of metastatic sites, %	
1	35
2	43
3	19
4	2
CA 19–9, median (range), U/mL^a^	602.8 (0.93–1000)
Biliary stent, %	1

^a^ CA 19–9 value above laboratory-defined upper limit of quantitation (1000 U/mL) is listed as 1000 U/mL

*CA 19–9* carbohydrate antigen 19–9, *KPS* Karnofsky performance status

### Efficacy Results

The initial dose administered to 15 patients in Part 1 was well tolerated; therefore, all patients in Part 2 were treated with *nab*-paclitaxel 125 mg/m^2^ plus gemcitabine 1000 mg/m^2^ qw 3/4. These 15 patients from Part 1 were included in Part 2. On the basis of combined results for Stages 1 and 2, the prespecified independently assessed ORR endpoint for Part 2 was met (35%; 95% CI, 24.8%–46.2%; Table 2). Although no complete responses were observed, there were 29 (35%) partial responses (PRs), and stable disease was achieved in 18 (22%) patients. Thirteen (16%) patients had progressive disease. The median DOR was 8.9 months (95% CI, 6.01–8.94), and the disease control rate was 55% (95% CI, 44.1%–66.3%; Table 2). Part 3 was not initiated per the study design (> 9 responses were observed in Part 2).

**Table 2:** Efficacy

Outcome^a^	*nab*-P + Gem *N* = 83
ORR, *n* (%)	29 (35)
CR	0
PR	29 (35)
SD, *n* (%)	18 (22)
PD, *n* (%)	13 (16)
Not evaluable, *n* (%)	15 (18)
No postbaseline assessment, *n* (%)	8 (10)
DCR, *n* (%)^b^	46 (55)
DOR, median (95% CI), months	8.9 (6.01–8.94)
OS, median (95% CI), months	9.2 (7.6–11.1)
OS rate, %	
3 months	89
6 months	70
9 months	53
12 months	30
15 months	15
Progression-free survival, median (95% CI), months	5.5 (5.29–7.16)

*CR* complete response, *DCR* disease control rate, *DOR* duration of response, *Gem* gemcitabine, *nab*-P *nab*-paclitaxel, *ORR* overall response rate, *OS* overall survival, *PD* progressive disease, *PR* partial response, *SD* stable disease

^a^Percents may not add up to 100 due to rounding

^b^Defined as the percentage of patients achieving objective tumor response or SD for ≥16 weeks

**Fig. 1:**
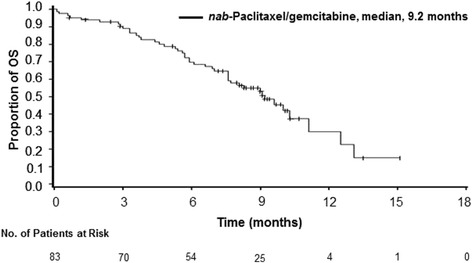
Kaplan-Meier curve of overall survival (OS) in Chinese patients with metastatic pancreatic cancer (MPC)

The median OS was 9.2 months (95% CI, 7.6–11.1; Fig. 1), and the 1-year OS rate was 30% (95% CI, 14%–47.6%); 15% of patients survived for ≥15 months. The median follow-up for OS was 8.9 months (range, 0.7–15.1 months). In an updated analysis approximately 1 year later, the median OS was 9.3 months, with a median follow-up of 14.6 months (range, 0.7–21.7 months). The 12-month OS rate was 32% in the follow-up analysis.

**Fig. 2:**
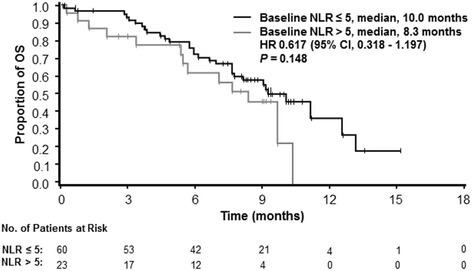
Kaplan-Meier curve of overall survival by neutrophil-to-lymphocyte ratio (NLR) in Chinese patients with metastatic pancreatic cancer (MPC)

**Fig. 3:**
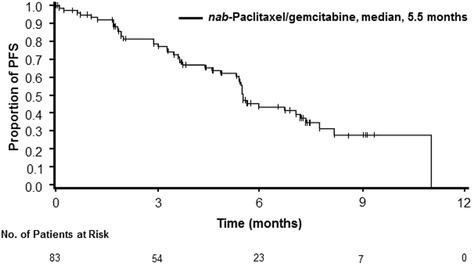
Kaplan-Meier curve of progression-free survival (PFS) in Chinese patients with metastatic pancreatic cancer (MPC)

Baseline NLR ≤ 5 was associated with a longer OS vs NLR > 5, although this difference was not significant (median, 10.0 vs 8.3 months; HR, 0.617; 95% CI, 0.318–1.197; *P* = 0.148; Fig. 2). Because the n value in the >5 NLR arm was small (*n* = 23), a separate analysis using the median NLR baseline value (3.7) was performed; baseline NLR ≤ 3.7 (*n* = 42) vs > 3.7 (*n* = 41) was also associated with a longer OS, but the difference was not significant (median, 10.0 vs 8.1 months; HR, 0.724; 95% CI, 0.398–1.319; *P* = 0.288). The median PFS was 5.5 months (95% CI, 5.29–7.16; Fig. 3).

### Treatment Exposure

For all patients, the median duration of treatment was 4.8 months, and the median number of treatment cycles was 5 (range, 1–12). Forty-nine percent of patients had ≥1 *nab*-paclitaxel dose reduction, and 51% of patients had ≥1 gemcitabine dose reduction, most due to AEs. The most common AEs leading to dose reduction of *nab*-paclitaxel and gemcitabine were thrombocytopenia (14% and 20%), neutropenia (14% and 16%), and leukopenia (11% and 13%), respectively. At least 1 *nab*-paclitaxel or gemcitabine dose delay occurred in 37% of patients. The median cumulative doses of *nab*-paclitaxel and gemcitabine were 1500 and 12,000 mg/m^2^, respectively. The median dose intensities of *nab*-paclitaxel and gemcitabine were 79 and 627 mg/m^2^/week, respectively. The median percentages of per-protocol dose of *nab*-paclitaxel and gemcitabine were 85% and 84%, respectively.

### Safety

Seventy-five percent of patients experienced ≥1 grade ≥ 3 AE (Table 3). The most common grade ≥ 3 AEs were leukopenia (35%), neutropenia (34%), anemia (15%), thrombocytopenia (10%), and fatigue (13%). Grade 3 peripheral neuropathy occurred in only 7% of patients (no grade 4 reported). Twenty-four percent of patients reported ≥1 serious treatment-emergent AE. Discontinuations due to AEs were relatively low (11%).

**Table 3:** Grade ≥ 3 treatment-emergent adverse events in ≥10% of patients

Grade ≥ 3 adverse events, *n* (%)	*nab*-P + Gem *N* = 83
Pts with at least 1 grade ≥ 3 AE	62 (75)
Hematologic AEs^a^	
Leukopenia	28 (35)
Neutropenia	27 (34)
Anemia	12 (15)
Thrombocytopenia	8 (10)
Nonhematologic AEs	
Fatigue	11 (13)

*AE* adverse event, *Gem* gemcitabine, *nab*-P *nab*-paclitaxel

^a^Based on laboratory values; *n* = 80 patients assessed

## Discussion

In this phase II study, the MPACT regimen (*nab*-paclitaxel 125 mg/m^2^ plus gemcitabine 1000 mg/m^2^) was efficacious and safe as first-line treatment of Chinese patients with MPC. Per protocol, the study did not progress to Part 3 because >9 responses were observed during Part 2 and the study was considered complete. Although no complete responses were observed in this study, 35% of patients had a partial response, and the median DOR was 8.9 months, indicating a durable response. The median OS was 9.2 months, and the OS rate at 1 year was 30% (9.3 months and 32%, respectively, in an updated analysis). The regimen appeared to be well tolerated in Chinese patients with MPC, and no new safety signals were identified compared with those observed in the MPACT population [7].

Efficacy results in this study of Chinese patients were comparable with those reported in MPC trials using the same *nab*-paclitaxel/gemcitabine regimen in Western countries and Japan [7, 8, 18]. In the MPACT population, treatment with this *nab*-paclitaxel/gemcitabine regimen resulted in a median OS of 8.7 months compared with 9.2 months in the Chinese population (Table 4) [8]. Similar to the findings of MPACT, Chinese patients with a baseline NLR ≤ 5 had a longer OS compared with those with a baseline NLR > 5. The ORR was 23% in MPACT and 35% in the Chinese population, although treatment resulted in a slightly longer DOR in the global study (11.1 months in the MPACT population and 8.9 months in the Chinese population) [19]. In both populations, the median PFS was 5.5 months.

**Table 4:** Efficacy Outcomes of *nab*-paclitaxel plus gemcitabine in MPACT and the Chinese study

Parameter	MPACT [7, 8, 19]	Chinese Study
n	431	83
OS, median, months	8.7	9.2
NLR ≤ 5	9.1	10.0
NLR > 5	5.0	8.3
PFS, median, months^a^	5.5	5.5
ORR, %^a^	23	35
DCR, %	48	55
DOR, median, months	11.1	8.9

*DCR* disease control rate, *DOR* duration of response, *NLR* neutrophil-to-lymphocyte ratio, *MPACT* Metastatic Pancreatic Adenocarcinoma Clinical Trial, *ORR* overall response rate, *OS* overall survival, *PFS* progression-free survival

^a^Independently assessed

Although data from other studies of Chinese patients treated with *nab*-paclitaxel/gemcitabine are limited, a phase I/II study evaluated 3 different doses of *nab*-paclitaxel (80 mg/m^2^, 100 mg/m^2^, and 120 mg/m^2^) in combination with gemcitabine 1000 mg/m^2^, both given weekly for 2 weeks in a 21-day cycle in Chinese patients with advanced pancreatic cancer [16]. In that study, the maximum tolerated dose was not met; however, in the 12 patients treated with *nab*-paclitaxel 120 mg/m^2^, the median OS and PFS were 12.2 and 5.2 months, respectively, and the ORR was 42%. Similar to the findings in our study, common grade 3/4 toxicities that were associated with the 120 mg/m^2^ dose included neutropenia (17%) and thrombocytopenia (8%), and grade 3/4 sensory neuropathy occurred in only 1 patient. In a trial of Japanese patients with MPC, outcomes of treatment with the *nab*-paclitaxel/gemcitabine MPACT regimen were also higher/longer compared with the outcomes in the MPACT population [18]. These findings further support the use of the MPACT regimen for the treatment of Asian patients with MPC.

In the current study, the most common treatment-emergent grade ≥ 3 AEs were leukopenia, neutropenia, anemia, thrombocytopenia, and fatigue. Similarly, the most common grade ≥ 3 AEs in MPACT were neutropenia, leukopenia, thrombocytopenia, anemia, fatigue, and peripheral neuropathy [7]. The incidence of peripheral neuropathy was one noteworthy difference between these two trials. In the MPACT population, 17% of patients experienced grade ≥ 3 peripheral neuropathy compared with only 7% of Chinese patients in this study. The definitive reasons for this are unclear, and many factors, such as ethnic differences or regional variations in treatments for neuropathy, could be involved [20, 21]; this would be an interesting topic to investigate in the future. In addition, *nab*-paclitaxel treatment modifications due to AEs were less frequent in the MPACT population compared with the Chinese population [7]. *nab*-Paclitaxel dose reductions occurred in 41% and 49% of patients in MPACT and the Chinese study, respectively.

Results from this phase II study in Chinese patients are positive; however, several factors must be considered to put the data in perspective. Although this was a bridging study to assess the safety and efficacy of *nab*-paclitaxel/gemcitabine in Chinese patients, one limitation was the homogeneous population. However, the impact of this limitation may have been addressed by the study’s multicenter sampling. In addition, efficacy was evaluated based on a single treatment arm rather than on a comparison of outcomes between 2 randomized groups. The results described here in Chinese patients are similar to those of the global MPACT study, though cross-trial comparisons should be interpreted with caution because of differences in factors such as patient population and usual supportive care. For example, in our study, a higher percentage of Chinese patients had a better baseline performance status (KPS of 90–100) than patients in the global MPACT population (70% vs 58%) [7]. Therefore, when comparing these 2 studies, it is possible that this difference could, in part, account for the improved efficacy outcomes observed in this study compared with the MPACT study. Further, although only the first 2 parts of the 3-part study design were executed, the null hypothesis was rejected as more than 9 of the 82 patients (planned sample size) responded. Although Part 3 would have provided more rigor to the overall statistical testing of *nab*-paclitaxel/gemcitabine vs gemcitabine in this disease setting, it would only have been triggered if sufficient activity was not observed vs known historical data in Part 2. Such adaptive trial designs are generally more efficient, requiring fewer patients to answer research questions. This unique study design was particularly beneficial and relevant in this disease setting and helped to avoid enrolling Chinese patients into an inferior treatment arm, as a large global study has established the significant clinical benefit of *nab*-paclitaxel/gemcitabine vs gemcitabine.

## Conclusion

The *nab*-paclitaxel/gemcitabine regimen used in MPACT was efficacious and well tolerated in Chinese patients with MPC, supporting the use of this combination regimen in this patient population.

## Abbreviations

AE: adverse event
DOR: duration of response
HR: hazard ratio
IV: intravenously
KPS: Karnofsky performance status
MPACT: Metastatic Pancreatic Adenocarcinoma Clinical Trial
MPC: metastatic pancreatic cancer
NLR: neutrophil-to-lymphocyte ratio
ORR: overall response rate
OS: overall survival
PFS: progression-free survival
PR: partial response
qw 3/4: the first 3 of 4 weeks
RECIST: Response Evaluation Criteria in Solid Tumors
